# Anti-Inflammatory Activities of Captopril and Diuretics on Macrophage Activity in Mouse Humoral Immune Response

**DOI:** 10.3390/ijms222111374

**Published:** 2021-10-21

**Authors:** Paweł Bryniarski, Katarzyna Nazimek, Janusz Marcinkiewicz

**Affiliations:** Department of Immunology, Jagiellonian University Medical College, 31-121 Krakow, Poland; katarzyna.nazimek@uj.edu.pl (K.N.); janusz.marcinkiewicz@uj.edu.pl (J.M.)

**Keywords:** antihypertensive therapy, diuretics, humoral immunity, hypotensive drugs, macrophages

## Abstract

Hypertension is accompanied by the over-activation of macrophages. Diuretics administered alone or in combination with hypotensive drugs may have immunomodulatory effects. Thus, the influence of tested drugs on mouse macrophage-mediated humoral immunity was investigated. Mice were treated intraperitoneally with captopril (5 mg/kg) with or without hydrochlorothiazide (10 mg/kg) or furosemide (5 mg/kg) by 8 days. Mineral oil-induced peritoneal macrophages were harvested to assess the generation of cytokines in ELISA, and the expression of surface markers was analyzed cytometrically. Macrophages were also pulsed with sheep red blood cells (SRBC) and transferred to naive mice for evaluation of their ability to induce a humoral immune response. Tested drugs increase the expression of surface markers important for the antigen phagocytosis and presentation. SRBC-pulsed macrophages from mice treated with captopril combined with diuretics increased the secretion of antigen-specific antibodies by recipient B cells, while macrophages of mice treated with hydrochlorothiazide or furosemide with captopril increased the number of antigen-specific B cells. Tested drugs alter the macrophage secretory profile in favor of anti-inflammatory cytokines. Our results showed that diuretics with or without captopril modulate the humoral response by affecting the function of macrophages, which has significant translational potential in assessing the safety of antihypertensive therapy.

## 1. Introduction

Macrophages mainly function as phagocytes that may present antigens to induce the adaptive humoral and cellular immune responses. However, these cells also play an important role in various other processes that are essential to maintain the body’s homeostasis, such as tissue repair and regeneration. On the other hand, dysregulated macrophage activity is observed in different disorders, including cardiovascular diseases [1]. Therefore, macrophages are currently considered promising cellular targets for selective drug delivery [2,3]. Furthermore, macrophages may bind and thus react to drug-derived bioactive compounds due to the expression of various receptors on both plasma membrane and intracellular membranes, as well as may sense and react to microenvironmental changes induced by the particular medication [2].

Hypertension is one of the most common chronic diseases in the elderly. The European Society of Cardiology and European Society of Hypertension (ESC/ESH), the International Society of Hypertension (ISH), and the National Institute for Health and Care Excellence define hypertension, using office-based blood pressure, as a systolic pressure ≥ 140 mmHg or diastolic pressure ≥ 90 mmHg [4,5,6]. Comorbidity of hypertension with other diseases, including heart failure, is often an indication to take diuretics combined with hypotensive drugs, for example from the group of angiotensin-converting enzyme inhibitors (ACEI). The latter medications are represented by captopril, which is also used in arterial hypertension, heart failure, myocardial infarction (treatment of clinically stable patients within 24 h of a heart attack), long-term prophylaxis of symptomatic heart failure in clinically stable patients (ejection fraction ≤ 40%) and in diabetic nephropathy. Captopril can cause some side effects, such as sleep disturbance, dizziness, shortness of breath and persistent cough, gastrointestinal discomfort, rash, and taste disturbances.

Furosemide represents the group of loop diuretics. In the Henle loop, it inhibits the reabsorption of chloride ions in the nephron. Furosemide is commonly used to treat acute left ventricular failure and exacerbations of chronic heart failure. Hydrochlorothiazide is a thiazide diuretic agent that acts in the final cortical segment of the ascending loop of Henle and the initial portion of the distal tubule. Hydrochlorothiazide is used in heart failure, edema, hypertension, hypocalcemia, hypercalciuria, or diabetes insipidus. It is also used in liver cirrhosis and some kidney disorders, such as nephrotic syndrome and acute glomerulonephritis. These drugs should be used with caution due to their quite common side effects, especially electrolyte disturbances that include hypokalemia, hyponatremia, hypochloremia, and also possible deficiencies of magnesium and calcium. The excessive intake of diuretics can lead to dehydration. Additionally, these drugs may cause hypotension (including orthostatic), skin rash, glycosuria, constipation, diarrhea, gastric irritation, nausea, vomiting, and acute kidney injury.

As mentioned above, macrophage functions could be directly or indirectly modulated by some medications. This in turn may either enhance or reduce the efficacy of the treatment or may cause some side effects. Interestingly, depletion of salt gradient within the kidney due to the administration of loop diuretics was recently shown to affect macrophage polarization in transplanted organs, which resulted in an increased risk of bacterial urinary tract infection [7]. On the other hand, recent studies in an animal model demonstrated that captopril may prevent the development of diabetic nephropathy by reducing the number of glomerular macrophages [8]. Due to the increase in the incidence of cardiovascular diseases and the decrease in the efficiency of the immune system with age, one can speculate that diuretics may additionally increase the risk of infections in such patients by affecting macrophages. However, very little is known about the direct influence of diuretics alone and combined with hypotensive drugs on the immune activity of macrophages. Answering this question should provide the basis for a better understanding of the immune-related effects of therapy with these drugs. This then should allow for more effective treatment of patients suffering from hypertension, or other cardiovascular diseases, without increasing the risk of infection resulting from the impairment of protective humoral immunity.

**Scheme 1:**
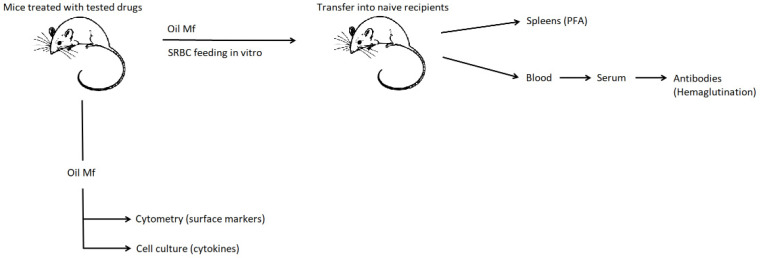
General scheme of experiments. Mice were administered with captopril with or without furosemide or hydrochlorothiazide for 8 days. On day 3, peritoneal exudate was induced with mineral oil. Peritoneal macrophages were harvested 5 days later and either cultured to assess cytokine secretion, analyzed cytometrically for surface marker’s expression, or pulsed with sheep red blood cells (SRBC) and transferred into naive mice, from which blood sera and spleens were collected a week later to measure the titer of SRBC-specific antibodies or to enumerate splenic SRBC-specific B cells in the plaque-forming assay (PFA), respectively.

We hypothesized that macrophage immune activity is modulated by diuretics and that combining them with the antihypertensive drug may influence this effect. Therefore, the current studies aimed at investigating the effect of furosemide and hydrochlorothiazide repeatedly administered to mice with or without captopril on macrophage activity in the induction of humoral immune response together with analysis of surface marker’s expression and secretion of cytokines (Scheme 1). Such a study model appears to have great translational potential, and thus the results could likely be implemented in clinical practice, personalized medicine especially, for the more effective and better medical care of patients with cardiovascular diseases [9,10].

## 2. Results

### 2.1. Drug Administration Influences the Induction of Humoral Immunity by SRBC-Pulsed Macrophages

Macrophages are involved in the induction of humoral immunity due to their ability to phagocytose corpuscular antigens, and then to present their determinants. In the current study, macrophages obtained from mice treated with captopril and/or with one of the diuretic drugs were pulsed with SRBC and then transferred into naive mice. The specific antibody titers were then measured in hemagglutination assay, and the number of antibody-producing cells in plaque-forming assay (PFA), in sera and spleens obtained from recipient mice 7 days after macrophage transfer.

**Figure 1:**
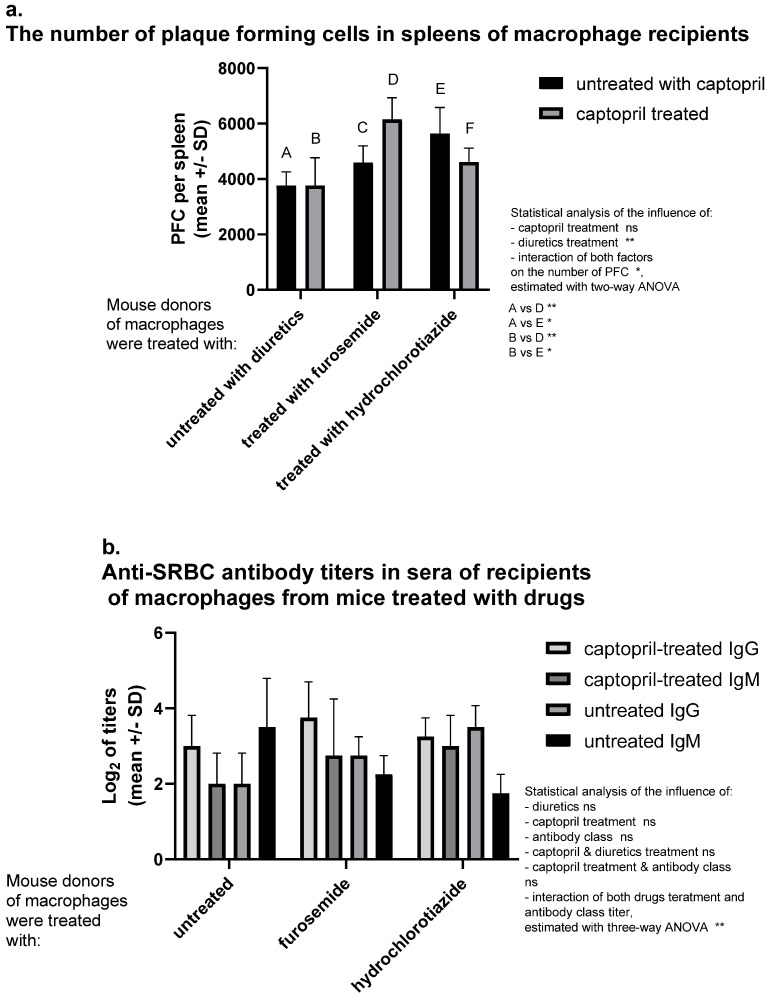
Captopril and diuretic drugs impact humoral immune response in mice. Donors of macrophages were treated intraperitoneally with captopril with or without furosemide or hydrochlorothiazide for eight days. Then, oil-induced peritoneal macrophages, after pulsing with sheep red blood cells (SRBC), were transferred intraperitoneally into recipients, from which sera and spleens were collected individually seven days later. (**a**) Number of plaque-forming cells (PFC) expressing SRBC-specific antibody-producing B cells in spleens of macrophage recipients was estimated through a plaque-forming assay and indicated as a mean number of PFC per spleen. (**b**) SRBC-specific antibody titers in sera of recipient mice were assessed through a direct hemagglutination assay and expressed as a mean value of log2 of antibody titer. Two-way or three-way ANOVA with Tukey post hoc test. * *p* < 0.05; ** *p* < 0.01; ns—not significant.

A significantly increased number of antibody-producing cells was observed in spleens of recipients of SRBC-pulsed macrophages from donors treated with hydrochlorothiazide. Captopril administered alone does not influence the number of antibody-producing cells. Interestingly captopril with hydrochlorothiazide have also no significant influence. In contrast, the administration of captopril with furosemide increases hemolytic plaque formation, i.e., activation of SRBC-specific B lymphocytes (Figure 1a). Captopril administration tends to enhance the antibody class switching process, which affects an increase in the concentration of IgG and a decrease in IgM concentration. Interestingly, furosemide seems to have an additive effect in contrast to hydrochlorothiazide (Figure 1b).

### 2.2. Drug Administration Alters the Expression of Macrophage Surface Markers

Macrophages play an antigen phagocytosing and presenting role in the induction of humoral immune response. Thus, as a next step, the expression of molecules involved in these processes on the macrophage surface was assessed cytometrically.

**Figure 2:**
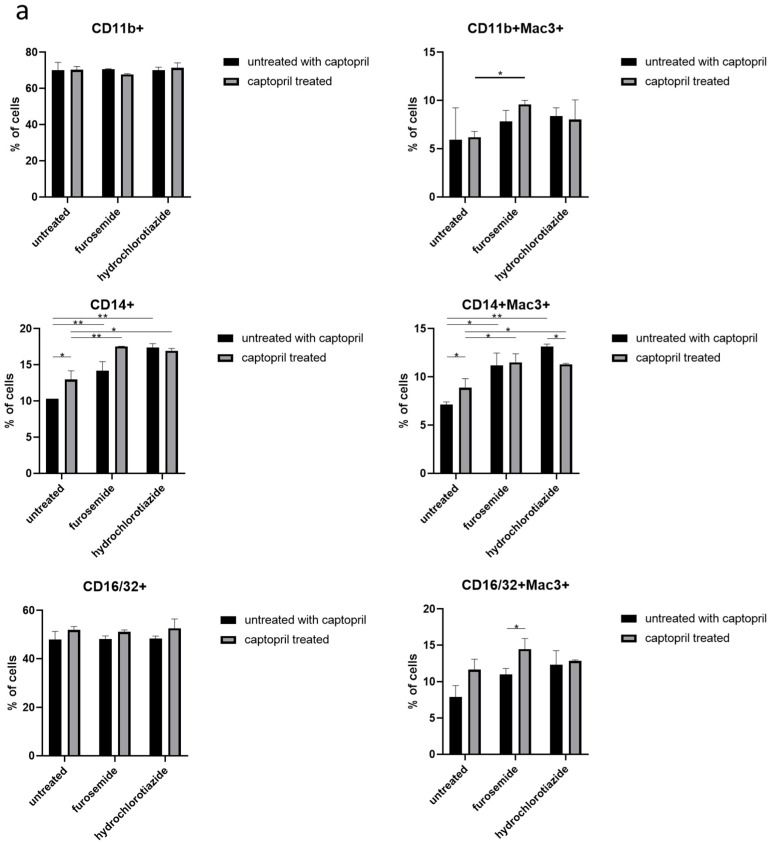

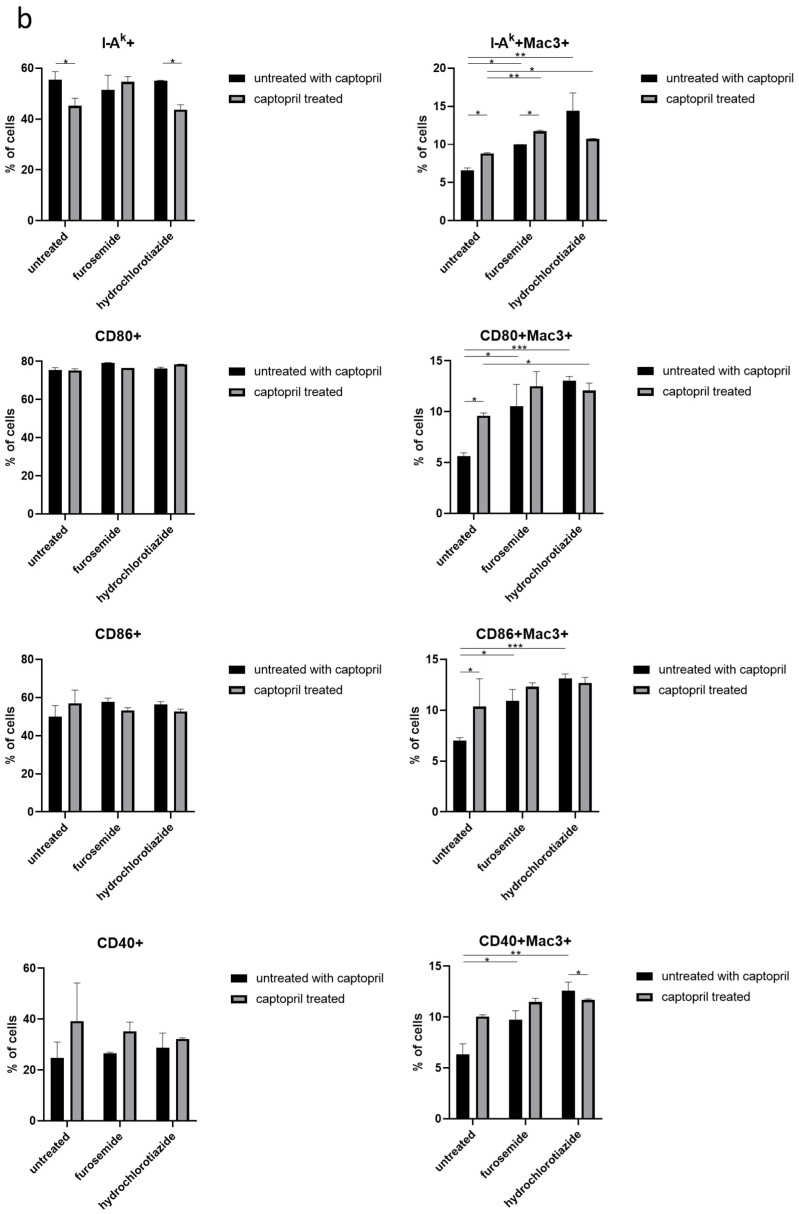
Captopril and diuretic drugs impact the expression of macrophage surface markers. (**a**) The level of expression of markers of phagocytosis (CD14, CD11b, CD16/32), and (**b**) antigen presentation, including I-A^k^ (MHC class II), CD80, CD86, and CD40, was cytometrically assessed on the surface of oil-induced peritoneal macrophages from mice treated for eight days with captopril and/or respective diuretic drug. Results were shown as a mean percentage (with SD) of macrophages expressing a particular marker within either the total population of analyzed macrophages or their Mac3+ subpopulation. Two-way ANOVA with Tukey post hoc test. * *p* < 0.05; ** *p* < 0.01; *** *p* < 0.005.

We observed that drug administration increases the expression of CD14 on both the total population and the Mac3+ subpopulation of macrophages. In addition, treatment of macrophage donors with captopril and furosemide increased the expression of CD11b receptor, and furosemide enhanced the captopril effect on CD16/32 expression, both on Mac3+ cells (Figure 2a).

On the other hand, captopril administration reduced the expression of I-A^k^ by the total population of macrophages obtained from mice treated only with this hypotensive drug or together with hydrochlorothiazide, but not with furosemide. Whereas, all drugs administered alone or in combination increased the expression of I-A^k^ by Mac3+ subpopulation of macrophages. A similar tendency was observed in the case of expression of costimulatory molecules, i.e., CD80, CD86, and CD40 by Mac3+ macrophages (Figure 2b).

### 2.3. Drug Administration Alters the Macrophage Secretion of Cytokines

Another important activity of macrophages inducing humoral immune response results from the secretion of cytokines. This macrophage activity was evaluated by culturing the cells and measuring cytokine concentrations in yielded supernatants. Since assayed drugs influenced the expression of CD14 that is activated by lipopolysaccharide (LPS), where indicated, macrophages obtained from drug-treated mice were stimulated with LPS during culture.

**Figure 3:**
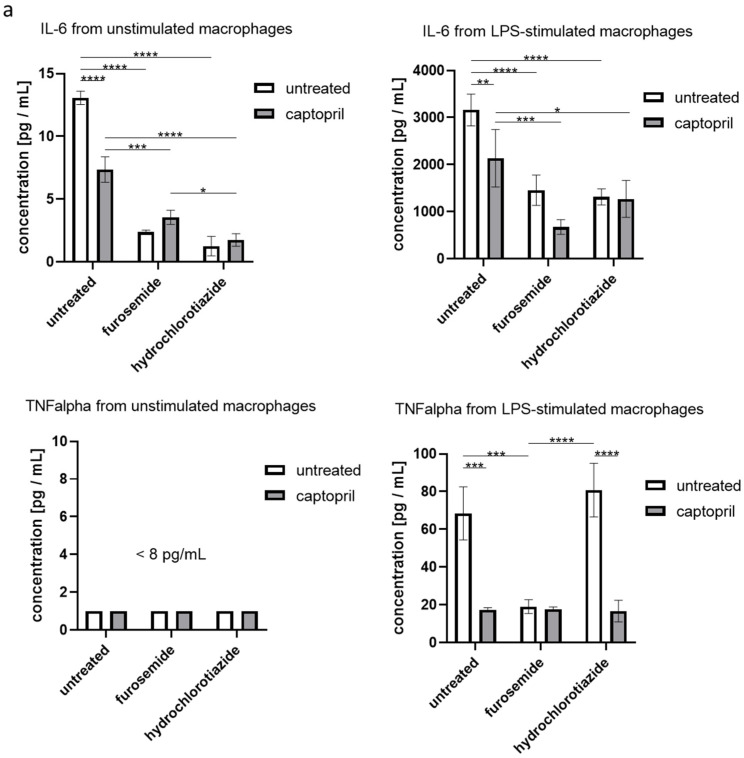

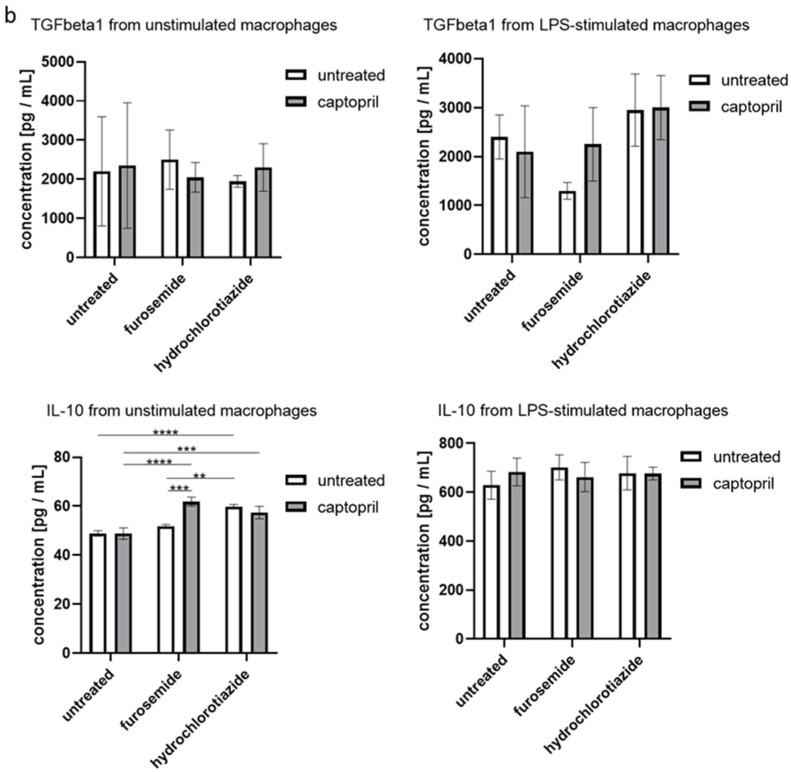
Captopril and diuretic drugs influence the secretion of cytokines by cultured macrophages. Oil-induced peritoneal macrophages from mice treated with captopril with or without respective diuretic drugs were cultured in standard conditions, in some cases after stimulation with LPS (200 ng). Enzyme-linked immunosorbent assay (ELISA) was used to measure (**a**) the concentration of IL-6, and TNFα in supernatants collected after 24 h of culture and (**b**) the concentration of IL-10 and TGF-β1 in supernatants collected after 48 h of culture. Concentrations were expressed as mean (+/− SD) per group. Two-way ANOVA with Tukey post hoc test. * *p* < 0.05; ** *p* < 0.01; *** *p* < 0.005; **** *p* < 0.001.

We found that administration of all tested drugs and their combinations strongly reduces the secretion of IL-6 by macrophages, regardless if cells were or were not re-stimulated with LPS in the culture. Furthermore, captopril administered with or without the diuretic drug decreased the LPS-stimulated secretion of TNFα (Figure 3a). On the other hand, neither captopril nor diuretics significantly influenced the release of TGFβ1 in both culture conditions, but slightly increased the secretion of IL-10 by macrophages that were not re-stimulated with LPS (Figure 3b).

## 3. Discussion

Current studies aimed at investigating the influence of captopril administered alone or combined with furosemide or hydrochlorothiazide on macrophage activity in humoral immune response in mice. Our results have significant meaning when assessing the risk of antihypertensive therapy.

Our current research findings demonstrated that captopril combined with a diuretic drug enhances the humoral immune response induction by macrophages by modulating their antigen-presenting and secretory activities. It is worth noting that medications may influence macrophage functions both by direct cell targeting via expressed receptors (including G-protein-coupled receptors) and by causing the microenvironmental changes that are then sensed by macrophages. Accordingly, both mechanisms could be involved in currently observed effects, especially that macrophages express angiotensin II receptors as well as can sense the ionic changes induced by diuretics.

Captopril has been reported to decrease the level of TNFα [11,12,13,14,15,16,17,18,19,20,21,22,23,24,25,26,27,28,29,30,31,32], reduce alveolar wall thickening and neutrophil infiltration in rats with induced acute lung injury [33]. Our research supports the results of other studies on the reduction of TNFα by ACEI. Captopril used in coronary artery disease and ischemic heart diseases reduced inflammation in vivo by lowering the level of IL-6 [11,12,18,19,22,34,35,36,37,38], and increasing the level of TGFβ since the macrophages are in vivo activated with drugs [37,38,39]. Other authors found that non-stimulated macrophages can express the undetectable concentration of TGFβ in cell culture supernatant [40]. In our study, we confirmed the observations of researchers regarding the effect of captopril on the concentration of IL-6. Interestingly, our experiments have shown that captopril slightly reduces the concentration of TGFβ, contrary to the experiments of the cited authors. Captopril reduced the concentration of the highly sensitive C-reactive protein (hs-CRP) [12,28,32]. Treatment with a high dose of captopril significantly reduced the number of Kupffer cells (liver macrophages) in the model of hepatic ischemia/reperfusion (I/R) injury in rats [13]. Captopril decreased the concentration of IL-1 beta and IL-6 in the different inflammatory rat models of renal hypertension, hepatic injury, myocarditis [11,12,35,41] as well as IL-8 reduction of human coronary artery endothelial cells [42]. On the other hand treatment with captopril promoted the production of anti-inflammatory IL-10 in various models of diseases induced in rats and mice as radiation-induced lung inflammation, biliary hepatitis, atherosclerosis, and hypertension as well as in normal mouse splenocytes [24,26,37,43,44]. According to our research, captopril slightly increased the production of IL-10, which is confirmed by the observations of other researchers. Captopril markedly attenuated macrophage accumulation and reduced the synthesis of radiation-induced interleukin-1β (IL-1β) [24,45]. Interestingly, the use of CHC (chronic high dose captopril) therapy increases the concentration of IL-1β and IL-6 in the plasma, i.e., completely different effects than in the case of normally used captopril doses [46]. In LPS-induced lung inflammation in rats, the treatment with captopril decreased the levels of IFN-γ, TGF-β1, and the IFN-γ/IL-4 ratio, contrary to the concentration of IL-4 [47]. Captopril increases the concentration of IL-22 in sera of patients suffer from hypertension and coronary artery disease which alleviates the clinical symptoms of the diseases [48]. In in vitro culture of human peripheral blood mononuclear cells, Captopril dose-dependently suppressed the IL-1β-induced synthesis of TNFα and the TNFα-induced synthesis of IL-1β [30].

In mice suffering from malaria, with the activation of splenic T cells an increase of 3–4 times of the expression of the CD69 marker, captopril provides an immunomodulating effect, restoring the values to control levels. Captopril also reduces the production of IFNγ and IL-17 by T CD4+ cells and abrogated the expression of the chemokine receptor (CCR2 and CCR5) and CD11 [49]. Our research has shown (unlike the cited authors) that treatment with captopril has practically no effect on CD11b expression on mouse macrophages. Furosemide slightly increases the expression of CD11b, which is increased by co-treatment with captopril. Hydrochlorothiazide acts similarly on the expression of CD11b by macrophages, however, the interaction with captopril does not affect the expression of that cluster of differentiation. Captopril administered to rats suffering from rheumatoid arthritis relieved congestion and swelling of the synovial membrane of the joint, significantly reduced synovial hyperplasia, infiltration of intra-articular inflammatory cells, and the degree of joint cartilage damage, which proves the strong anti-inflammatory nature of the drug [20].

Strong anti-inflammatory properties of captopril are shown by its influence on the reduction of IL-2 production, and IL-2Rα expression by mouse CD8+ T lymphocytes and IFNγ by human T CD3+ CD28+ T cells [15,38,42]. Captopril also reduces the production of both subtypes of IL-12 (p40 and p70) by human peripheral blood mononuclear cells (PBMC) [42] and down-regulates the immune cells infiltrating arterial atherosclerotic lesions by down-regulating the C-C chemokine receptor 9 (CCR9) induced in atherosclerosis-prone apolipoprotein E (ApoE) deficient mice [50].

Moreover, captopril in a dose-dependent manner increases antioxidant markers (total thiol, superoxide dismutase, and catalase), so we can conclude that this drug reduces oxidative stress, apart from earlier mentioned anti-inflammatory properties [18,22].

The concentration of proinflammatory cytokines, i.e., TNFα and IL-6 measured in supernatants of human peripheral blood mononuclear cells of a patient with chronic heart failure were inhibited in a dose-dependent manner by furosemide treatment [19,51].

Furosemide decreased the concentration of TNFα in SARS-CoV-2 infected patients [45], and together with IL-6 in the pre-eclamptic placenta [46], children with mild asthma [47], patients with respiratory disorders [48], patients with urosepsis [49], or patients with acute decompensated heart failure [52]. Alleviation of IL-1beta concentration after furosemide treatment was observed in a newborn with neonatal nephrotic syndrome [50,53], patients with neuroinflammation disorders, COVID-19, and women in pregnancy [54,55,56]. In our studies, we have shown that furosemide reduces TNFα alone and in combination with captopril (however adding captopril to furosemide does not affect TNFα concentration), and alone and in combination with captopril reduces IL-6 levels (however, adding captopril enhances the cytokine reduction). Furosemide enhances the expression of the anti-inflammatory IL-1RA in patients with neuroinflammation disorders or in people who suffered from SARS-CoV-2 infection when furosemide is administered by inhalation [57,58]. Furosemide reduces the expression of CD86 markers of inflamed microglial cells [57]. Our research has shown (in contrast to the cited authors) that treatment with furosemide increases the expression of CD86, which is slightly enhanced by co-treatment captopril with furosemide. Captopril increases CD86 expression compared to control, as does hydrochlorothiazide, adding captopril to a thiazide-like diuretic does not affect the expression of this cluster of differentiation. Furosemide promotes the phagocytic activity of macrophages [57]. There is also observed the furosemide activated switching of M1 type macrophages to anti-inflammatory M2 phenotype [57,58]. Administration of furosemide decreases procalcitonin blood concentration as a marker of microbial inflammation, suggesting its anti-inflammatory properties [59]. Furosemide reduces the production of the anti-inflammatory IL-10 in preeclamptic placentas and placentas of normal gestation [55,60]. According to our research, furosemide alone and in combination with captopril slightly increases the production of IL-10 by macrophages, which calls into question the existing findings in this case. Furosemide did not show any significant effect on IL-2 secretion of PBMC in patients with heart failure [25]. and reduces the concentration of IL-8 in PBMC patients suffered from asthma and respiratory tract diseases [56,61]. Furosemide reduces the concentrations of IL-6 and TNFα intracellularly, which has been confirmed by cell cytometry [56]. During our experiments, we checked how furosemide and the combined drug (furosemide + captopril) affect the concentration of TGFβ. This is revealing and interesting as we have not found information in the available literature to have ever researched it. Our research shows that furosemide significantly reduces the concentration of TGFβ, but adding captopril to it practically eliminates the suppressive effect of furosemide.

Hydrochlorothiazide is a diuretic drug used for the treatment of hypertension. Spontaneously hypertensive rats (SHR) when treated with hydrochlorothiazide increases the splenic expression of CD62L hi (naive) and CD62L lo (memory) T cells, but reduces expression of CD62L(-) effector T cells in comparison to the control group of SHR. The percentage of regulatory T and Th CD25+ splenocytes were not affected with hydrochlorothiazide treatment of SHR [62].

Hydrochlorothiazide reduces the concentrations of TNFα and IFNγ in supernatants from cell culture of hydrochlorothiazide treated splenocytes obtained from SHR [62]. Our studies have shown that hydrochlorothiazide slightly increases the concentration of the pro-inflammatory cytokine TNFα, however, adding captopril causes a significant reduction in the production of TNFα. Hydrochlorothiazide reduces the expression of TGFβ in cardiac tissue in rat experimental models of ischemic heart failure and myocardial infarction [63,64]. In congestive heart failure after myocardial infarction, hydrochlorothiazide improves heart remodeling by reducing the levels of proinflammatory cytokines and inhibiting the TGFβ signaling pathway [64]. As our research shows, hydrochlorothiazide increases the concentration of TGFβ, interestingly, adding captopril to it does not change the cytokine concentration compared to its level after adding hydrochlorothiazide. Hydrochlorothiazide therapy does not alter CD3 (+), CD4 (+), CD8 (+), and Th17 T cells, but reduces Treg lymphocytes in female rat kidneys with spontaneous hypersensitivity [65]. According to the previous research, hydrochlorothiazide did not express any anti-inflammatory activity based on peripheral blood polymorphonuclear leukocytes analysis from patients with essential hypertension and human neutrophil studies [45,66]. In our study, which was tested for the first time, we showed that hydrochlorothiazide used alone and with captopril has practically no effect on the concentration of IL-10. An uncharted problem is the effect of hydrochlorothiazide and the combined drug (hydrochlorothiazide + captopril) on the concentration of the cytokine IL-6. We have shown that hydrochlorothiazide significantly reduces the production of this cytokine, and the addition of captopril to a thiazide-like diuretic does not affect the concentration of the produced cytokine.

In our work, which is innovative, we present research on the effects of captopril, furosemide, and hydrochlorothiazide on the expression of CD14, CD16/32, CD40, Ak (MHC), and CD80, which have not been studied so far. Our studies showed that adding each drug increases CD14 expression, with hydrochlorothiazide having the strongest effect. Captopril increases CD16/32 expression, as do furosemide and hydrochlorothiazide. The addition of captopril to furosemide increases the expression of this marker, while the addition of ACEI to the thiazide-like diuretic has practically no effect on the expression of CD16/32. In the case of CD40, we observe an increase in its expression with the addition of each drug, most strongly in the case of hydrochlorothiazide. Our studies have shown that each of the drugs increases the expression of I-A^k^ (MHC), and while the addition of captopril to furosemide shows an additive effect, the addition of ACEI to a thiazide-like diuretic reduces the strong positive effect of hydrochlorothiazide. In the case of the last tested cluster of differentiation (CD80), we observe an increase in its expression with the use of each of the drugs, but while the addition of ACEI to a loop diuretic potentiates its stimulating effect, the addition of ACEI to a thiazide-like diuretic does not affect the increased expression of CD80 by hydrochlorothiazide.

Another significant achievement of our work is the demonstration of the effect of the tested drugs on the formation of early humoral response cells and the demonstration that all drugs administered, except captopril administered alone, increase the induction of antibody-producing B cells (Figure 1a). Our experiments also prove that the use of the tested drugs has a beneficial effect on the maturation of the humoral response, which has not been proven so far (Figure 1b).

The latter observations show experimental evidence that diuretics combined with captopril may induce anti-inflammatory effects associated with the reduced secretion of proinflammatory cytokines, which could alleviate the inflammatory-induced complications in hypertensive patients and patients suffering from other inflammatory diseases (metabolic syndrome). Moreover, our findings exert important clinical significance by suggesting the possible improvement of humoral immune response to vaccination in patients treated with the assayed drugs in comparison to others with dysregulated inflammatory responses accompanying the uncontrolled/untreated hypertension.

## 4. Materials and Methods

### 4.1. Mice

In all experiments, 10–12-week-old male mice (24 ± 2 g) of the inbred CBA strain from the breeding unit of the Department of Immunology, Jagiellonian University Medical College, Krakow, Poland were used according to the guidelines of the 1st Local Ethics Committee (approval no. 81/2017 and 434/2020). Mice had unlimited access to autoclaved food and water. The general experimental design is shown in Scheme 1.

### 4.2. Antihypertensive Drug Administration

All drugs were from (Sigma-Aldrich, St. Louis, MO, USA). Hydrochlorothiazide and furosemide aliquots were firstly dissolved in 100 µL of dimethyl sulfoxide (DMSO, Sigma-Aldrich, St. Louis, MO, USA), and then in 9.9 mL of 0.9% sodium chloride (Chempur, Piekary Slaskie, Poland), while captopril was dissolved in 0.9% sodium chloride alone. Mice were administered intraperitoneally (i.p.) with captopril solution in a daily dose of 5 mg/kg, for 8 consecutive days. Where indicated, mice simultaneously received i.p. injections of either hydrochlorothiazide in a daily dose of 10 mg/kg or furosemide in a daily dose of 5 mg/kg.

### 4.3. Induction and Harvest of Peritoneal Exudate Macrophages

On day 3 of drug administration, mice were administered i.p. with mineral oil, and peritoneal exudates were collected 5 days later by washing the peritoneal cavity with ice-cold Dulbecco’s phosphate-buffered saline (DPBS) containing heparin (5 U/mL, Polfa, Warszawa, Poland) [9]. After washing, cell preparations (containing more than 96% of non-specific esterase-positive cells [41]) were used in the following assays as peritoneal macrophages obtained from drug-treated donors and untreated control mice.

### 4.4. Activation and Measurement of Humoral Immune Response to Sheep Red Blood Cells (SRBC)

Macrophages were pulsed with SRBC (Graso Biotech, Starogard Gdanski, Poland) by 20-min incubation at 37 °C in a ratio of ten SRBC per one macrophage. After the osmotic shock, SRBC-pulsed macrophages were transferred intraperitoneally into naive recipients (5 × 10^6^ cells per mouse), and 7 days later blood and spleens were collected individually from each mouse to measure the serum antibody titer and the number of antibody-producing cells, respectively. Serum titers of total IgM and IgG anti-SRBC antibodies as well as IgG antibodies (in sera pre-incubated with 0.15 M 2-mercaptoethanol from Sigma-Aldrich, St. Louis, MO, USA) were measured in direct hemagglutination assay [9] and expressed as a log2. In addition, SRBC-specific IgM antibody titers were calculated by subtracting IgG titers from the total titers, individually for each mouse. In parallel, single-cell suspensions in RPMI1640 (Lonza Group Ltd., Basel, Switzerland) were prepared by homogenizing each, previously weighted spleen separately. Then, splenocyte suspensions were individually incubated with 1% SRBC suspension in the presence of guinea pig complement for 90 min at 37 °C to assess the number of plaque-forming cells (PFC) in a plaque-forming assay (PFA) performed by a slide technique. Each splenocyte sample was evaluated in triplicates, and the averaged results were expressed as the number of PFC per spleen [9,67,68].

### 4.5. Macrophage Culture, and the Measurement of Cytokine Concentration

Macrophages from control or drug-treated mice were standardly cultured at 37 °C and 5% CO_2_, at a concentration of 2 × 10^6^ cells per well in 2 mL of RPMI1640 with 5% fetal bovine serum (FBS, Biowest, Nuaillé, France). Macrophages in some wells were stimulated with lipopolysaccharide (LPS, 200 ng per well, BIO-Whittaker, Walkersville, MD, USA). The resulting supernatants were collected for evaluation of IL-6, and tumor necrosis factor-alpha (TNFα) concentrations after 24 h, and for IL-10 and transforming growth factor-beta 1 (TGF-β1) measurement after 48 h of the culture. Cytokine concentrations were measured in supernatants stored at −80 °C, in enzyme-linked immunosorbent assay (ELISA), according to manufacturer procedures. The following OptEIA™ kits were used: mouse TNF ELISA Set (catalog No 558534), mouse IL-6 ELISA Set (catalog No 555240), mouse IL-10 ELISA Set (catalog No 555252), all from (BD Biosciences, San Diego, CA, USA). In addition, TGF-β1 concentration was measured with the use of mouse TGF-beta1 Platinum ELISA Test (catalog No BMS608/4) from (eBioscience Inc., San Diego, CA, USA).

### 4.6. Cytometric Analysis of Macrophages

In cytometric analysis, fluorescein isothiocyanate (FITC)-conjugated rat anti-mouse Mac-3 monoclonal antibody (mAb), phycoerythrin (PE)-conjugated rat anti-mouse I-Ak, CD80, CD86, CD40, CD11b, CD14, CD16/32 monoclonal antibodies (mAb) (BD Pharmingen, San Diego, CA, USA) were used.

Apart from assessing CD16/32 expression, peritoneal macrophages, freshly isolated from naive or drug-treated mice, were incubated with mAb of 2.4G2 clone to block Fc receptors. Afterward, cells were incubated with FITC-conjugated and PE-conjugated mAbs for 40 min on ice in darkness. After washing, macrophages were analyzed on a FACSCalibur flow cytometer (BD Bioscience, San Jose, CA, USA) for the expression of the abovementioned markers.

### 4.7. Statistical Analysis

All experiments were performed two to four times, and representative results are shown in the figures. Statistical analysis was performed in GraphPadPrism 8 (GraphPad, San Diego, CA, USA) using one-, two-, or three-way analysis of variance (ANOVA) with a post hoc RIR Tukey test. All ANOVA assumptions were validated before analysis. In two-way ANOVA, the captopril treatment was assigned as column factor, and treatment with dehydrating drugs was a row factor, while in one-way ANOVA drug treatment was the main factor. *p* < 0.05 was considered as a minimum level of significance.

## 5. Conclusions

Our current research findings demonstrated that repeated administration of captopril combined with a diuretic drug (furosemide or hydrochlorothiazide) augments the antigen-presenting activity of mouse macrophages in the induction of humoral immune response. Interestingly, this is associated with an increased expression of surface receptors and markers involved in the process of antigen binding, phagocytosis, and presentation, including CD14, MHC class II and costimulatory molecules. However, assayed drugs reduce the secretion of proinflammatory cytokines by macrophages, and slightly enhance the release of IL-10, which seems to promote the anti-inflammatory activity of captopril administered with the diuretic drug.

## Data Availability

All data are included within the manuscript.
